# Osteoarthritis of the hip joint in elderly patients is most commonly atrophic, with low parameters of acetabular dysplasia and possible involvement of osteoporosis

**DOI:** 10.1007/s11657-017-0325-4

**Published:** 2017-03-22

**Authors:** Yasuhiro Ishidou, Kanehiro Matsuyama, Daisuke Sakuma, Takao Setoguchi, Satoshi Nagano, Ichiro Kawamura, Shingo Maeda, Setsuro Komiya

**Affiliations:** 1 Department of Medical Joint Materials, Graduate School of Medical and Dental Sciences, Kagoshima University, 8-35-1 Sakuragaoka, Kagoshima, 890-8520 Japan; 2 The Near-Future Locomotor Organ Medicine Creation Course (Kusunoki Kai), Graduate School of Medical and Dental Sciences, Kagoshima University, Kagoshima, Japan; 3 Department of Orthopedic Surgery, Graduate School of Medical and Dental Sciences, Kagoshima University, Kagoshima, Japan

**Keywords:** Osteoarthritis, Osteoporosis, Acetabular dysplasia, Aging

## Abstract

**Abstract:**

**Summary:**

As elderly patients with hip osteoarthritis aged, acetabular dysplasia parameters decreased (Sharp’s angle, acetabular roof obliquity angle, and acetabular head index) and the incidence of the atrophic type increased. Vertebral body fracture was more frequent in the atrophic type, suggesting the involvement of osteoporosis at the onset of hip osteoarthritis.

**Introduction:**

Osteoarthritis (OA) is associated with increased bone formation at a local site. However, excessive bone resorption has also been found to occur in the early stages of OA. Osteoporosis may be involved in the onset of OA in elderly patients. We conducted a cross-sectional radiographic study of patients with hip OA and examined the association between age and factors of acetabular dysplasia (Sharp’s angle, acetabular roof obliquity angle, and acetabular head index) as well as the osteoblastic response to determine the potential involvement of osteoporosis.

**Methods:**

This study included 366 patients (58 men, 308 women) who had undergone total hip arthroplasty for the diagnosis of hip OA. We measured the parameters of acetabular dysplasia using preoperative frontal X-ray images and evaluated each patient according to Bombelli classification of OA (hypertrophic, normotrophic, or atrophic type).

**Results:**

As the patients aged, the parameters of acetabular dysplasia decreased. The incidence of the atrophic type of OA was significantly higher in older patients. Vertebral body fractures were more frequent in the atrophic type than in the other types. Additionally, the index of acetabular dysplasia was lower in the atrophic type. By contrast, the hypertrophic type was present in relatively younger patients and was associated with an increased index of acetabular dysplasia.

**Conclusion:**

In elderly patients with hip OA, the parameters of acetabular dysplasia decreased and the incidence of the atrophic type increased as the patients aged. The frequency of vertebral body fracture was high in patients with the atrophic type, suggesting the involvement of osteoporosis in the onset of hip OA.

## Introduction

Osteoarthritis (OA) is associated with increased bone formation at a local site, whereas osteoporosis involves decreased bone formation with increased bone resorption, resulting in reduced bone mass [1]. Therefore, an inverse relationship has been identified between OA and osteoporosis [2]. However, excessive bone resorption characteristic of osteoporosis has also been found to occur in the early stages of OA [3], drawing attention to the functional unit consisting of articular cartilage and subchondral bone [4]. Molecular crosstalk between cartilage and subchondral bone contributes to the pathogenesis of OA [5–8]. Microstructural damage to the subchondral bone and subsequent bone loss by increased remodeling is associated with the pathogenesis of early-stage OA [9, 10]. Anti-osteoporosis drugs such as bisphosphonates are candidate therapeutic drugs with which to prevent the progression of OA [11, 12]. However, the curative effects of these drugs are controversial. The pattern of the biological bone response in OA can be classified as hypertrophic or atrophic based on the presence or absence of osteophytes, respectively [13–15]. As a result of the heterogeneity of OA, differences in radiographic patterns may be useful in elucidating differences in a patient’s reaction to an anti-osteoporosis drug.

OA is a mechanically induced disease affected by both genetic and acquired factors [4]. Acetabular dysplasia is one of the anatomical risk factors for OA [16, 17], and genetic polymorphism associated with the risk of developmental dysplasia of the hip was recently identified [18]. OA associated with acetabular dysplasia secondary to genetic factors is predicted to have a relatively early onset. In Japan, developmental acetabular dysplasia of the hip is reportedly among the most common causes of hip OA [19, 20]. Meanwhile, the incidence of acetabular dysplasia is lower in elderly patients [21]. The incidence and prevalence of OA after 50 years of age are higher in women than in men, suggesting that estrogen participates in the development of OA [22]. These findings suggest that the etiologic factors of OA in elderly patients may differ from those in young patients and that osteoporosis is involved in the onset of OA in elderly patients. However, few studies have investigated this hypothesis.

We conducted a cross-sectional radiographic study in patients with hip OA and examined the characteristic of hip OA in the elderly, including the association between parameters of acetabular dysplasia of the hip and age as well as the osteoblastic response, to examine the potential involvement of osteoporosis.

## Patients and methods

### Patients

A total of 441 consecutive patients who underwent total hip arthroplasty for OA during the 2-year period from January 2013 to December 2014 at Kagoshima University Medical and Dental Hospital and other participating institutions were evaluated. Radiographs and information about the diagnosis were collected from medical records. We excluded cases of posttraumatic OA, OA after infection or osteonecrosis, and previous osteotomy. The study finally included 366 patients (58 men, 308 women; 63 were aged 30–59 years, 90 were aged 60–69 years, 129 were aged 70–79 years, and 84 were aged ≥80 years; there were no significant differences in the sex ratio within each age group).

### Radiographic measurement

**Fig. 1:**
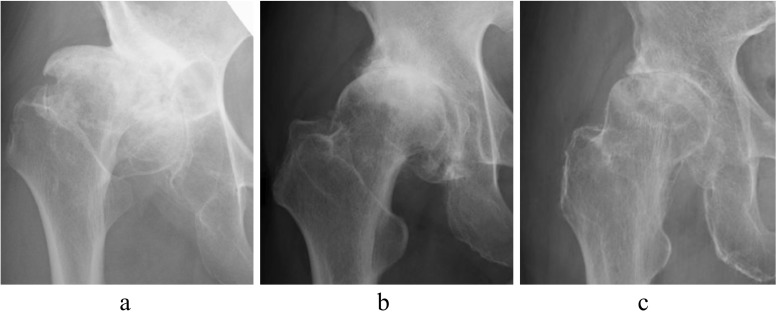
Radiographs of OA according to the Bombelli classification. Typical radiographs of hypertrophic (**a**), normotrophic (**b**), and atrophic (**c**) OA

We measured the parameters of acetabular dysplasia using frontal X-ray images of bilateral hip joints taken preoperatively. Sharp’s angle [23], the acetabular roof obliquity (ARO) angle [24], and the acetabular head index (AHI) [25] were used as reproducible radiographic parameters of acetabular dysplasia [19, 26]. The center edge (CE) angles showed low reproducibility, as the femoral head center could not be precisely determined due to severe deformities associated with end-stage OA. We also classified the patients according to Bombelli classification of OA (hypertrophic type, normotrophic type, and atrophic type) suggesting a biological osteoblastic response [27]. Bombelli classification was carefully evaluated as described by Saito et al. [28]. We show typical radiographs of hypertrophic, normotrophic, and atrophic OA in Fig. 1. The Bombelli classification was independently assessed by three orthopedic surgeons, with required agreement by at least two. When one judgment differed, the determination of the other two was adopted. The kappa score for intra- and interobserver agreement was 0.83 and 0.77–0.93, respectively.

We also evaluated 250 patients (33 men, 217 women; <60 years of age, 43 patients; 60–69 years of age, 50 patients; 70–79 years of age, 93 patients; and ≥80 years of age, 64 patients) for the presence or absence of vertebral compression fracture and compared these findings with the hip joint findings obtained using Bombelli classification. Vertebral compression fractures were assessed by one senior spinal surgeon using a semiquantitative technique described by Genant et al. [29].

### Statistical analysis

Single regression analysis was performed for statistical analysis of the correlation between the parameters of acetabular dysplasia and age. The Steel–Dwass test was used as a nonparametric multiple comparison test in each age group. The Steel–Dwass test was also used as a nonparametric multiple comparison test for the results of Bombelli classification. The significance level was set at *p* < 0.05. Statistical analyses were performed using Statcel3 software (OMS, Saitama, Japan).

## Results

**Fig. 2:**
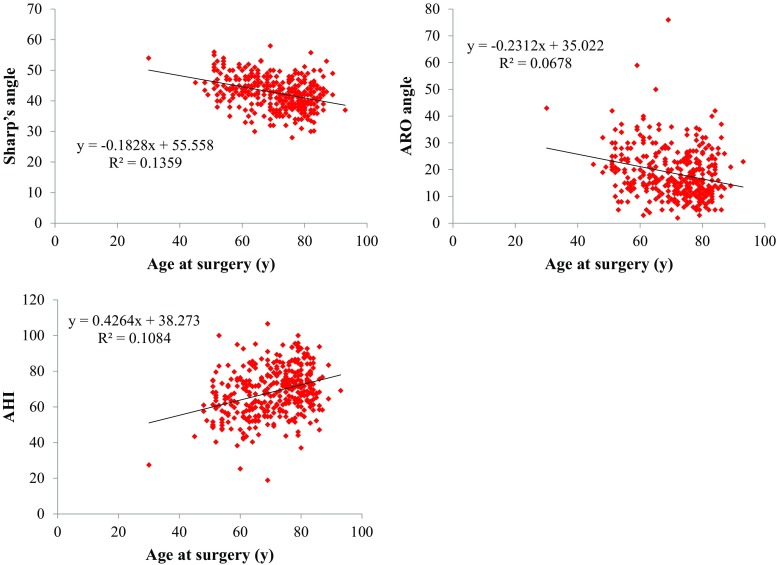
The correlation between the acetabular index and age. Single regression analysis was performed for statistical analysis of the correlation between the parameters of acetabular dysplasia and age. *ARO* acetabular roof obliquity, *AHI* acetabular head index

**Fig. 3:**
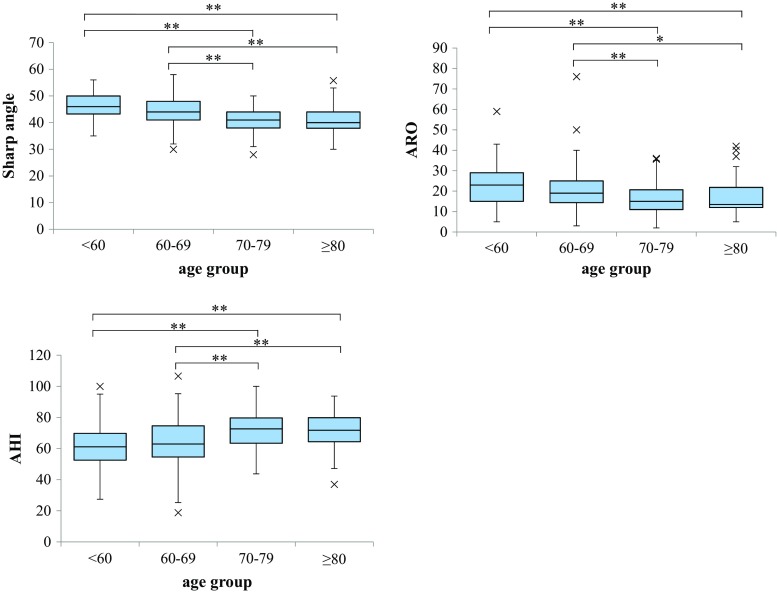
The comparison of acetabular index in age groups. The Steel–Dwass test was used as a nonparametric multiple comparison test in each age group. *ARO* acetabular roof obliquity, *AHI* acetabular head index. *p < 0.05; **p < 0.01

The mean Sharp’s angles were 46.1°, 43.9°, 41.1°, and 41.0° in patients aged <60, 60–69, 70–79, and ≥80 years, respectively. The corresponding ARO angles were 23.0°, 20.6°, 16.3°, and 17.0°, respectively, and the corresponding mean AHIs were 61.8, 64.8, 71.9, and 72.1%, respectively (Table 1). As the patients became older, Sharp’s angle and the ARO angle decreased significantly (*R* = −0.3686, *p* < 0.01 and *R* = −0.2603, *p* < 0.01, respectively) and the AHI increased significantly (*R* = 0.3292, *p* < 0.01) (Fig. 2). Namely, as the patients became older, the parameters of acetabular dysplasia decreased. In a comparison according to age group, Sharp’s angle and the ARO angle were significantly smaller in patients aged 70–79 and ≥80 years. The AHI was significantly larger in patients aged 70–79 and ≥80 years (Fig. 3).

**Table 1:** The parameters of acetabular dysplasia

	Age group (years)	<60	60–69	70–79	≥80
	Number	63	90	129	84
Sharp’s angle	Average	46.1	43.9	41.1	41.0^a^
	SD	4.66	5.26	4.41	4.84
	SE	0.587	0.555	0.388	0.528
	Min	35.0	30.0	28.0	30.0
	Median	46.0	44.0	41.0	40.0
	Max	56.0	58.0	50.0	55.8
ARO angle	Average	23.0^a^	20.6^a^	16.3^a^	17.0^a^
	SD	9.86	10.5	7.47	8.21
	SE	1.24	1.10	0.658	0.896
	Min	5.00	3.00	2.00	5.00
	Median	23.0	19.0	15.0	13.5
	Max	59.0	76.0	36.0	42.0
AHI	Average	61.8	64.8	71.9	72.1
	SD	13.0	14.9	12.0	11.4
	SE	1.64	1.57	1.05	1.24
	Min	27.5	18.9	43.8	37.0
	Median	61.2	63.0	72.7	71.8
	Max	100.0	106.6	100.0	93.7

*SD* standard deviation, *SE* standard error, *Min* minimum, *Max* maximum, *ARO* acetabular roof obliquity, *AHI* acetabular head index

^a^Nonnormal distribution

**Fig. 4:**
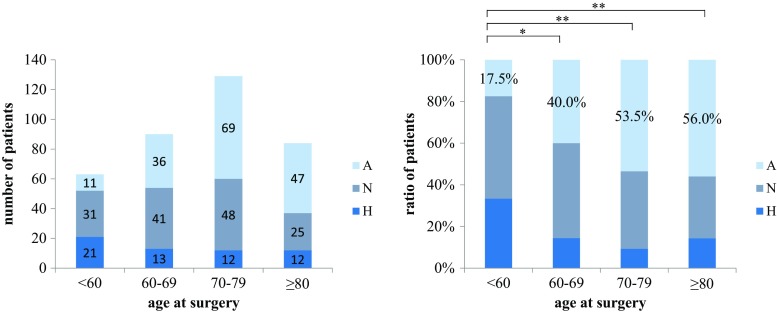
Bombelli classification in age groups. The Steel–Dwass test was used as a nonparametric multiple comparison test in each age group. *H* hypertrophic type, *N* normotrophic type, *A* atrophic type. *p < 0.05; **p < 0.01

**Fig. 5:**
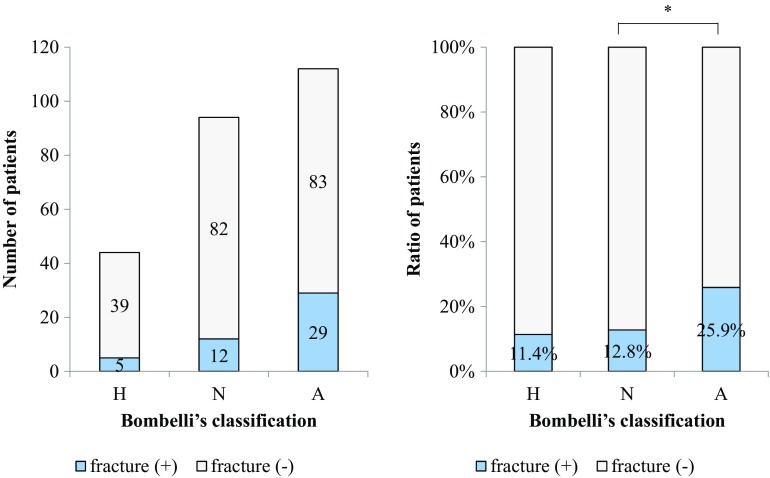
The incidence of vertebral body fracture. The Steel–Dwass test was used as a nonparametric multiple comparison test for the results of Bombelli classification. *H* hypertrophic type, *N* normotrophic type, *A* atrophic type. *p < 0.05

The frequency of the atrophic type of OA according to Bombelli classification was 17.5, 40.0, 53.5, and 53.5% in patients aged <60, 60–69, 70–79, and ≥80 years, respectively, showing an increase with age (Fig. 4). The frequency of vertebral body fracture was 11.4% in the hypertrophic type, 12.8% in the normotrophic type, and 25.9% in the atrophic type, with the atrophic type showing the highest frequency (Fig. 5).

**Fig. 6:**
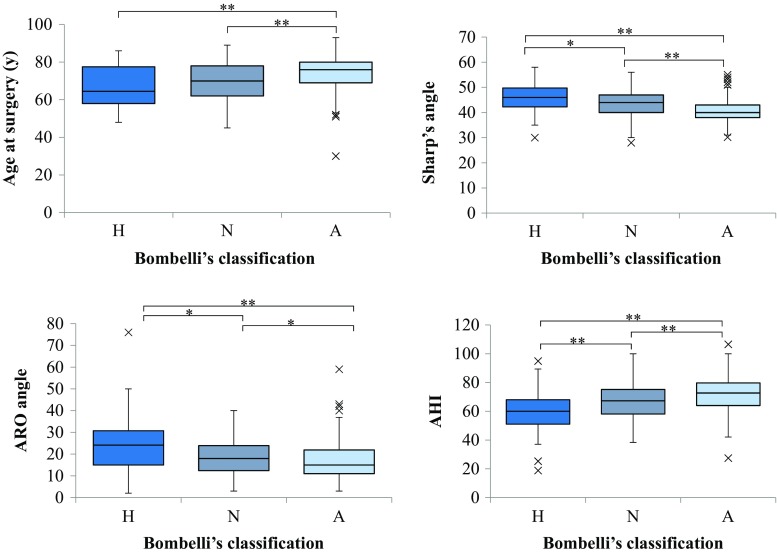
The comparison of acetabular index in Bombelli classification. The Steel–Dwass test was used as a nonparametric multiple comparison test for the results of Bombelli classification. *H* hypertrophic type, *N* normotrophic type, *A* atrophic type, *ARO* acetabular roof obliquity, *AHI* acetabular head index. *p < 0.05; **p < 0.01

The atrophic type of OA occurred in patients of a significantly high age. Sharp’s angle and the ARO angle in the atrophic type were smaller than those in the other types. The AHI in atrophic type was larger than that in the other types. Namely, the parameters of acetabular dysplasia decreased in the atrophic type. On the other hand, the hypertrophic type occurred in patients of a relatively low age, in whom the parameters of acetabular dysplasia increased (Fig. 6).

## Discussion

We conducted a radiographic study of patients with hip OA requiring total hip arthroplasty and evaluated the correlation between parameters of acetabular dysplasia and aging. Additionally, we estimated the osteoblastic response in patients with OA according to Bombelli classification. As our patients become older, the parameters of acetabular dysplasia decreased and the incidence of the atrophic type of OA increased.

Acetabular dysplasia is an anatomical risk factor for hip OA. Reijman et al. [16] reported that acetabular dysplasia (center-edge angle of <25°) was associated with a 4.3-fold increased risk for incident radiographic hip OA. Jingushi et al. [19] reported that Sharp’s angles of >45° or 50° had significantly increased odds ratios for OA (5- or 65-fold greater, respectively) in comparison to <40°.

However, a recent epidemiological study of hip OA in Japan demonstrated that the incidence of acetabular dysplasia was lower in elderly patients [21]. Lane et al. [30] also reported that the association between acetabular dysplasia and OA in white women aged ≥65 years was not determined. In the present study, the indices of acetabular dysplasia in the elderly age groups (70–79 and ≥80 years of age) were significantly lower than those in the younger age groups. Additionally, these acetabular dysplasia parameters were negatively correlated with age.

Anatomical abnormalities such as acetabular dysplasia produce abnormal or incongruous loading on articular cartilage for long periods, resulting in cartilage degeneration at a comparatively younger age [13, 31]. Therefore, the incidence of OA secondary to acetabular dysplasia is high in comparatively young individuals, but low in those who are older. OA secondary to acetabular dysplasia has onset in middle age, and induces a brisk osteoblastic response due to the abnormal mechanical load, resulting in hypertrophy.

Meanwhile, primary OA in hip joints without anatomical abnormalities will maintain essentially normal cartilage under normal conditions of loading at a younger age, but nonmechanical etiological factors such as osteoporosis may cause cartilage failure at a later age. Therefore, primary OA without acetabular dysplasia shows higher incidence in older individuals, and induces a weak osteoblastic response to osteoporosis, resulting in atrophy.

OA is generally characterized by cartilage degradation, new bone spur formation, and osteosclerotic changes of the subchondral bone. However, there is little osteoblastic activity in the atrophic type of OA, so no osteophytes are observed. The atrophic type of OA is associated with a higher risk of joint destruction [32], more rapid disease progression [33], lower mineral density, and higher risk of osteoporotic fracture than other types of OA [34]. Our data showed that the incidence of atrophic OA was higher as the patients became older. In addition, the incidence of vertebral fracture in the atrophic type was higher than in other types of OA. Schnitzler et al. [32] also reported a high prevalence of vertebral fracture in the atrophic type of OA. These views support the concept that osteoporosis is involved in the etiology of the atrophic type of OA.

However, a limitation of our study was the absence of bone mineral density measurements. Because this osteoporosis study was retrospective, we could not collect sufficient data on bone mineral density. Therefore, we inferred that osteoporosis was potentially related to the etiology of atrophic OA by showing the high incidence of vertebral compression fractures in atrophic OA. A prospective epidemiological study to clarify this concept is necessary.

Osteoporosis may induce microstructural damage to the subchondral bone by increased remodeling, resulting in the onset of OA [9, 35]. A prospective randomized study of alendronate treatment for hip OA showed significant improvement in pain but no significant prevention of structural disease progression [36]. More effective curative results may be expected if the patients are limited to the atrophy type strongly influenced by osteoporosis. Microstructural damage to the subchondral bone induces inappropriate crosstalk between osteoblasts and chondrocytes [6–8]. It is probably important to begin treatment for osteoporosis before such events occur. When planning the optimal OA treatment strategy, it is important that we consider the age of onset and heterogeneity of the etiology.

Various factors, including genetic predisposition, sex, age, obesity, physical activity, joint injury, joint malalignment, and abnormal joint shape, affect the subchondral bone integrity [37]. We statistically demonstrated in this radiographic study that the atrophic type of OA has a predominantly older age at onset and is affected by osteoporosis, whereas the hypertrophic type of OA has a predominantly younger age at onset and is affected by acetabular dysplasia. Because the atrophic type of OA is not accompanied by anatomical abnormalities, it is difficult to predict the onset of atrophic OA before cartilage degradation. No serum biomarkers that are able to demonstrate differences between the atrophic and hypertrophic types of OA have been identified [14]. Additionally, the older onset of atrophic OA may be affected not only by osteoporosis but also by mild acetabular dysplasia [38]. Some studies have reported that the patterns of osteoblastic reaction in the osteoarthritic hip were not related to the general bone mineral density [34, 39]. The uncertain definition of the atrophic type of OA and the lack of appropriate biomarkers are problematic.

This cross-sectional study showed that atrophic OA increases with aging. Various factors other than anatomical abnormalities affect the pathogenesis of OA. Osteoporosis may be a contributor, but this remains controversial. Pathological classification in individual OA cases may be helpful for selection of drug treatment. Further research is needed to understand the difference in the types of OA and heterogeneity of the etiology.

## Conclusion

In elderly patients with hip OA, the parameters of acetabular dysplasia decreased and the atrophic type increased as patients become older. The frequency of vertebral body fracture was higher in the atrophic type, suggesting the involvement of osteoporosis in the onset of hip OA.
